# Successful salvage therapy with Daptomycin for osteomyelitis caused by methicillin-resistant *Staphylococcus aureus *in a renal transplant recipient with Fabry-Anderson disease

**DOI:** 10.1186/1476-0711-11-6

**Published:** 2012-03-11

**Authors:** Ennio Polilli, Tamara Ursini, Elena Mazzotta, Federica Sozio, Vincenzo Savini, Domenico D'Antonio, Michelino Barbato, Augusta Consorte, Giustino Parruti

**Affiliations:** 1 Microbiology and Virology Unit, Pescara General Hospital, Pescara, Italy; 2 Clinic of Infectious Diseases, G. D'Annunzio University, Chieti, Italy; 3 Infectious Disease Unit, Pescara General Hospital, Pescara, Italy; 4 Orthopedics Unit, Ortona Hospital, Ortona, Italy; 5 Infectious Disease Unit, Pescara General Hospital, Via Fonte Romana, 8, Pescara 65124, Italy

**Keywords:** Methicillin-resistant *Staphylococcus aureus*, Osteomyelitis, Daptomycin, Salvage therapy, Antibiotic therapy

## Abstract

Daptomycin is licensed in adults for the management of *Staphylococcus aureus *methicillin-resistant infections, including bone and skin complicated infections. We describe for the first time its use in a renal transplant recipient for Fabry-Anderson Disease with right heel osteomyelitis. The patient was unresponsive to first-line Teicoplanin and second-line Tigecycline, whereas he was successfully treated with third-line Daptomycin monotherapy at 4 mg/Kg/qd for 4 weeks. Local debridement was performed in advance of each line of treatment.

## Background

Vancomycin, the first-line antibiotic choice for bone and joint infections caused by grampositive bacteria, has been reported with declining rates of efficacy against methicillin resistant *Staphylococcus aureus *(MRSA) isolates [1]. The treatment of osteomyelitis, septic arthritis and prosthetic joint infections with Vancomycin can therefore be difficult, often requiring prolonged administration [2-5]. The reduced susceptibility of *S. aureus *to Vancomycin may be partially due to biofilms, facilitating bacterial persistence [6-8]. Daptomycin, presently considered as a reliable alternative to the class of glycopeptides in these conditions [9], was recently used successfully, alone or in combination, for osteomyelitis caused by gram-positive pathogens including MRSA, unresponsive to other antibiotics [10]. Dosing of Daptomycin, however, and its ability to penetrate into inflamed target tissues are still a matter of controversy [11]. In this report we describe for the first time a case of right hell osteomyelitis caused by MRSA and successfully treated with third-line Daptomycin in a renal transplant recipient for Fabry-Anderson Disease (FAD).

## Case presentation

A 38-year-old patient, diagnosed with FAD in 1990, was put on hemodialysis since 1999 due to end-stage renal insufficiency and received a kidney transplant in 2007. Renal function did not recover immediately after renal transplantation, and sequential renal biopsies documented early rejection. For this reason, the patient was put on high dose oral Cyclosporine (150 mg twice daily) and was treated with high dose steroids without benefit. Everolimus (2.5 mg/daily) was therefore added and steroids tapered. Renal function improved 4 months after transplantation; hemodialysis was continued until that time. Due to relapsing decreases in renal function after each attempt to interrupt Everolimus, the association of these drugs could not be stopped ever since, although Cyclosporine could be reduced to 100 mg twice daily after 1 year. Levels of both drugs were frequently monitored, falling in the therapeutic range throughout follow-up without further adjustments. Additional drugs prescribed after transplantation were: Cardioaspirin, 100 mg/daily; Allopurinol 100 mg/daily; Carvedilol 25 mg/daily, Furosemide 25 mg/daily, Replagal^® ^(Agalsidase α) 3 times/weekly until hospitalization. During hemodialysis, he suffered several traumatic and ischemic bone lesions; a Dual-energy X-ray absorptiometry (DEXA) scan, performed in 2006, revealed diffuse osteopenia, in spite of intact parathyroid glands and normal parallel parathormone levels.

**Figure 1:**
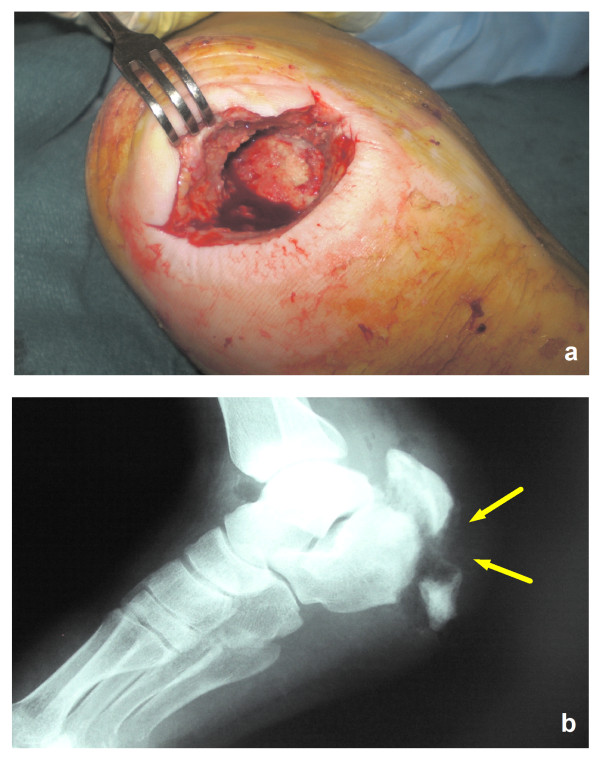
**a) Ulcerative lesion of the right heel; b) Standard Rx scan of the right heel at patient entry**.

**Figure 2:**
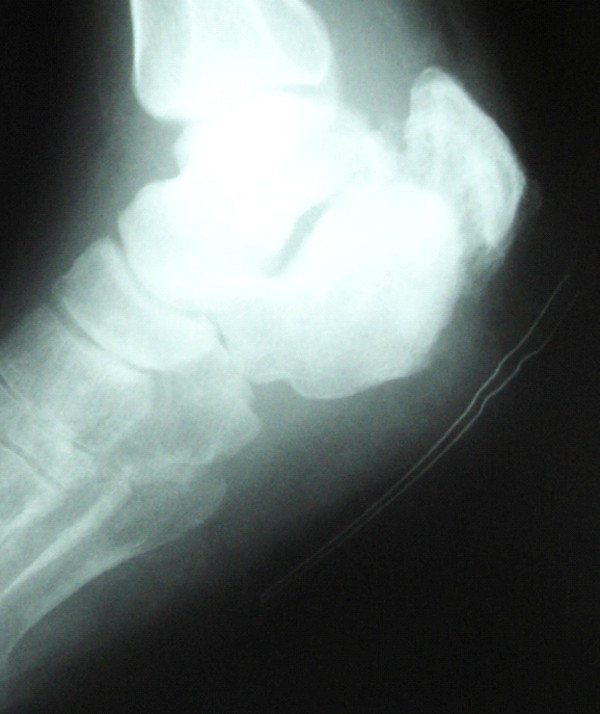
**Standard Rx scan of the right heel at end of treatment**.

In March, 2008, the patient was assisted in our ward due to a large pulmonary infiltrate and treated with i.v. Amoxicillin/Clavulanate and Fluconazole. The next month he was hospitalized in the orthopedic ward of Ortona Hospital (Chieti, Italy) because of an ulcerative lesion at his right heel with redness, swelling and the presence of necrotic tissue (Figure 1a). He was apyretic, with normal white and red cell counts and near-normal renal function tests, the estimated glomerular filtration rate ranging between 56 and 83 mL/min/1.73 m^2 ^during hospitalization, as calculated using the CKD-EPI formula. Standard Rx scans of his right foot documented a detachment of a wide fragment of his cortical spongiosa in the lateral malleolus (Figure 1b). Cultural examination of wound essudates did not yield any isolate. The patient underwent extensive debridement of necrotic tissues and was put on hyperbaric oxygen therapy and Teicoplanin 200 mg i.v. for 4 weeks, without benefit. The dose of Teicoplanin was decided by the assisting orthopedists at that time, the fear of renal overload causing patent underdosing. In July, 2008, the patient was again evaluated in the Infectious Disease Unit of Pescara General Hospital for persistent fever and localized pain. Inflammation indexes were remarkably altered. A second debridement procedure was requested; cultures of debris grew several *Streptococcus agalactiae *isolates. To avoid renal overload, treatment with i.v. Tigecycline, 50 mg daily twice daily, together with Metronidazole, 500 mg three times daily, was prescribed. The patient was still assisted at the orthopedic ward of Ortona Hospital after consultation; a double daily access was arranged for him as outpatient, and the prescription of Metronidazole reduced to 500 mg twice daily. A slight local improvement was noted, but relapsing fever ensued after 30 days of treatment. ESR rose to 112 mm, CRP to 85 mg/L. A focused CT scan of his right heel confirmed the presence of a fracture of his right calcaneus with fragmentation and irregular thickening of fracture edges, tarsal bone loss, gaseous components in neighboring soft tissues, abnormal spacing between calcaneus and cuboid. A few days later, the patient underwent a third debridement procedure. Culture specimens grew MRSA isolates, sensitive to Vancomycin (MIC = 0.5 mg/L) and Daptomycin. Based on these findings, i.v. Daptomycin (4 mg/kg daily) was started, and administered for 4 weeks. CRP levels had dropped to 56 mg/L at this time. CPK levels during treatment were frequently monitored and found unmodified. At the end of treatment, deep palpation of the lesion did not cause pain; accompanying skin lesions had resolved; liver and renal function tests were normal, ESR was 50 mm and CRP 6,5 mg/L. Standard Rx scans and focused control tomography of the heel documented a reduction of bone resorption at affected segments, indicating effective control of osteomyelitis (Figure 2). The patient was discharged, and antibiotic therapy was continued at home with oral Trimetoprim/Sulfamethoxazole (1 double strength tablet twice daily) and Doxycycline (100 mg twice daily) for 4 additional weeks, with frequent monitoring of hematological parameters, immunosuppressant drugs and renal function tests. No signs of toxicity were noted; the estimated value of GFR ranged between 63 and 85 mL using the CKD-EPI formula. At the end of treatment, the patient could walk with the support of crutches. In February, 2009, a complete normalization of inflammation indices was documented.

## Discussion

Bone and joint discomfort is a common problem after transplantation. It is now well known that skeletal abnormalities, especially osteopenia with subsequent fractures, may develop following kidney transplantation [12,13], osteomyelitis being one of the complications of fractures [14]. The complex impairment of bone metabolism due to FAD and long lasting hemodialysis in our patient may well have played a role in generating his recalcitrant infection [15]. At our best knowledge, here we report for the first time that Daptomycin in monotherapy at low dose (4 mg/Kg/daily) was safe and efficacious as a third line treatment for MRSA osteomyelitis of the heel in a renal transplant recipient on long-term immune suppression with Cyclosporine and Everolimus. Appropriate debridement of the lesion was performed in this patient before each of his treatment lines. No isolate was generated after the first procedure, and *Staphylococcus *osteomyelitis was postulated as a complication of his previous pneumonia. Vancomycin sensitivity of *S. aureus *was preserved until recently [16]; Vancomycin, however, has poor bone penetration and was unable to sterilize bone infections in animal models [17,18]. In spite of recent reports on its efficacy in human osteomyelitis at higher doses, the use of Vancomycin in our transplanted patient with ensuing full dose immunosuppressants was worrisome in the absence of a microbiological isolate, as the high recommended trough levels for Vancomycin (15-20 mg/L) in osteomyelitis might indeed have harmed the renal graft [19]. Low dose Teicoplanin was then preferred. Tigecycline plus Metronidazole were chosen after the second debridement because of the isolates of *Streptococcus agalactiae *obtained at that time. The etiologic role of these bacteria, however, may be questioned also in view of treatment failure. MRSA infection was finally documented by specimens cultured at the third debridement procedure. Monotherapy with Daptomycin for the following 4 weeks, at the cautious dose of 4 mg/Kg/daily, was preferred to Vancomycin. Long term administration of Linezolid, which tested equally effective and could have been given on an outpatient basis as oral therapy, was reckoned as potentially associated with worrisome side effects in this patient; furthermore, it is not permitted for treatment over 21 days [20]. Daptomycin is presently considered as an attractive alternative to glycopeptides in these cases, because of its ability to penetrate into inflamed subcutaneous adipose tissue and bones; in our patient it induced a rapid bone and wound recovery, with reversal of inflammation indexes [11]. The potency of Daptomycin has been demonstrated against a wide range of aerobic and anaerobic Gram-positive bacteria, including MRSA, glycopeptide-intermediate *S. aureus*, methicillin-resistant Coagulase-negative Staphylococci, and Vancomycinresistant *Enterococcus *(VRE) in bacteremias and endocarditis [21,22], skin and skin structure infection [23] and bone infections [10]. Daptomycin is safe with any state of renal function and dosing guidelines are available for hemodialysis patients [24] as well as for ICU patients with acute renal failure [25]. The characteristics and outcomes of patients with osteomyelitis who were treated with Daptomycin at doses ranging between 3.2 and 7.5 mg/Kg/daily (median initial dose 5.6 mg/kg) were retrospectively evaluated in the CORE 2004 database. Among clinically evaluable patients with osteomyelitis, 63% were cured. In 48% of them, Daptomycin was given concurrently with other antimicrobial agents; MRSA accounted for 45% of the identified pathogens. In another series, a 94% success rate was observed in patients treated with Daptomycin alone [26]. Although renal transplant recipients are most intensely immunosuppressed early in the posttransplantation period, immunosuppressive therapy could not be tapered in our patient over the past 3 years and during treatment. Therefore, the efficacy of Daptomycin, in conjunction with the repeated local debridement, may have played a major role.

## Conclusions

Our experience adds to the large body of evidence that Daptomycin is effective and does not carry a relevant risk of renal damage in fragile patients as those with impaired renal function, renal transplant recipients or patients on nephrotoxic drugs. It could be effectively and safely used for MRSA osteomyelitis in a renal transplant recipient with recovered renal function. Higher dosing at 6 to 8 mg/kg/daily should be cautiously tested in the event of life-threatening infections, with tight monitoring of ensuing nephrotoxicity due to immunosuppressive agents [27,28].

### Consent

Written informed consent was obtained from the patient for publication of this Case report and any accompanying images. A copy of the written consent is available for review by the Editor-in-Chief of this journal.

## Abbreviations

MRSA: Methicillin resistant *Staphylococcus aureus*; FAD: Fabry-Anderson Disease; DEXA: Dual-energy X-ray absorptiometry; VRE: Vancomycin-resistant *Enterococcus*.

## Competing interests

The authors declare that they have no competing interests.

## Authors' contributions

EP, GP, AC and DD ideated this case-report and did most of the writing, supported by TU. VS, EM and MB have been involved in drafting the manuscript. FS has made substantial contributions to acquisition of data. GP has made substantial contributions to conception of the case redaction, have given final approval of the version to be published. All authors read and approved the final manuscript.
